# The predictors of 3- and 30-day mortality in 660 MERS-CoV patients

**DOI:** 10.1186/s12879-017-2712-2

**Published:** 2017-09-11

**Authors:** Anwar E. Ahmed

**Affiliations:** Associate Professor, College of Public Health and Health Informatics, King Saud bin Abdulaziz University for Health Sciences, Riyadh, Saudi Arabia

**Keywords:** Mortality, MERS-CoV, Elderly, Camels, Saudi Arabia

## Abstract

**Background:**

The mortality rate of Middle East Respiratory Syndrome Coronavirus (MERS-CoV) patients is a major challenge in all healthcare systems worldwide. Because the MERS-CoV risk-standardized mortality rates are currently unavailable in the literature, the author concentrated on developing a method to estimate the risk-standardized mortality rates using MERS-CoV 3- and 30-day mortality measures.

**Methods:**

MERS-CoV data in Saudi Arabia is publicly reported and made available through the Saudi Ministry of Health (SMOH) website. The author studied 660 MERS-CoV patients who were reported by the SMOH between December 2, 2014 and November 12, 2016. The data gathered contained basic demographic information (age, gender, and nationality), healthcare worker, source of infection, pre-existing illness, symptomatic, severity of illness, and regions in Saudi Arabia. The status and date of mortality were also reported. Cox-proportional hazard (CPH) models were applied to estimate the hazard ratios for the predictors of 3- and 30-day mortality.

**Results:**

3-day, 30-day, and overall mortality were found to be 13.8%, 28.3%, and 29.8%, respectively. According to CPH, multivariate predictors of 3-day mortality were elderly, non-healthcare workers, illness severity, and hospital-acquired infections (adjusted hazard ratio (aHR) =1.7; 8.8; 6.5; and 2.8, respectively). Multivariate predictors of 30-day mortality were elderly, non-healthcare workers, pre-existing illness, severity of illness, and hospital-acquired infections (aHR =1.7; 19.2; 2.1; 3.7; and 2.9, respectively).

**Conclusions:**

Several factors were identified that could influence mortality outcomes at 3 days and 30 days, including age (elderly), non-healthcare workers, severity of illness, and hospital-acquired infections. The findings can serve as a guide for healthcare practitioners by appropriately identifying and managing potential patients at high risk of death.

## Background

In September of 2012 [1]^,^ the Middle East Respiratory Syndrome Coronavirus (MERS-CoV) was initially identified in humans in a 60-year-old Saudi male. Since then, outbreaks of MERS-CoV have been noted in Saudi Arabia. Although the number of new cases has dropped tremendously, to date the Saudi Ministry of Health continues to receive cases. The World Health Organization (WHO) routinely receives these cases in situational reports, and on June 23, 2016, WHO warranted reviewing unusual patterns, continued surveillance, and applying control measures [2]. The clinical and the epidemiological outcomes of MERS-CoV have been major public health concerns and a burden on the healthcare systems, and have become a top priority in Saudi Arabia [3]. It has been reported that Saudi Arabia has the largest MERS population throughout the world, followed by the Republic of Korea and the United Arab Emirates [2]. Saudi Arabia hosts Hajj every year, and it is a potential zone for rapid respiratory infection, particularly MERS-CoV re-emergence, since more than 2 million pilgrims arrive annually in Makkah to conduct Hajj for a period of 5 days. As of November 17, 2016, and according to WHO, since its emergence in Saudi Arabia, 1826 laboratory-confirmed MERS-CoV cases have been identified worldwide, resulting in a total of 649 deaths related to MERS-CoV [2]. This results in an overall MERS-CoV mortality rate of 35.5% in worldwide cases.

According to the Saudi Ministry of Health, between 2012 and November 14, 2016, a total of 1480 confirmed MERS-CoV cases have been reported publicly: 616 deaths, 855 cases recovered, and 9 cases under treatment. This results in an overall mortality rate of 41% on reported cases in Saudi Arabia. However, a number of other studies in Saudi Arabia have reported MERS-CoV with widely varying mortality rates. Assiri reported a 60% mortality rate on a sample 47 cases [4], while another study reported a mortality rate of 42% [5]. Two other studies reported a mortality rate of 40% [6, 7], and Coleman reported a mortality rate of 38% [8]. The reported mortality rates in Saudi Arabia tend to be higher than in other countries [9]. A study in Jordan reported a mortality rate of 22% in MERS cases [10], while two studies in South Korea reported 19% [11] and 20.4% [12] mortality rates in MERS cases.

Several studies reported that elderly patients [13–19], patients with comorbidities [14, 18, 19], and men [19, 20] are at high risk of death and of developing MERS-CoV complications. However, other studies noted that the frequency of deaths was less in men [21] and in healthcare workers [13, 18, 20, 21]. Little data has been collected on risk-standardized mortality rates and multivariate risk factors of 3- and 30-day mortality in the MERS-CoV population. Per the author knowledge, a single study used Poisson regression to identify risk factors for overall mortality and severe MERS-CoV disease [18]. The current study established MERS-CoV mortality measures at 3 days and 30 days. The study aimed to determine the 3- and 30-day survival rates and their risk factors on a large MERS-CoV population in Saudi Arabia.

## Methods

### Data sources

The Saudi Ministry of Health receives new confirmed cases from hospitals across the kingdom. The Saudi Ministry of Health provides daily situational reports on new confirmed cases and provides a routine update on developments to the community and the media through the Command and Control Center (CCC) hotline and the website. Since its emergence, the MERS-CoV data was made publicly available on the Saudi Ministry of Health website directory at http://www.moh.gov.sa/en/ccc/pressreleases/pages/default.aspx. As the study data are publicly available online, it does not require Institutional Review Board (IRB) oversight and approval. The author analyzed publicly available national-level data on MERS-CoV cases and deaths that were reported from December 2, 2014 until November 12, 2016 by the Saudi Ministry of Health. The author used data from this period because a standard data collection was used during this time to obtain relevant demographic, clinical, and epidemiological information from each MERS-CoV patient. The data has been extracted by the study author and reviewed and entered by a research assistant. Data quality has been verified and assessed by both the author and the research assistant. The available data is anonymous, with no patient identifiers, and provides information on age, gender, Saudi nationality (Yes/No), whether healthcare worker (Yes/No), pre-existing illness (Yes/No), severity of illness (Yes/No), and death (Yes/No). The reported severity of illness of these patients may not reflect the up-to-date final medical condition status. The ages of patients were classified into two groups: elderly ≥60 years and <60 years old. MERS-CoV source of infections was investigated and reported as camels, household contact, hospital-acquired infection, and unknown infection source. MERS-CoV data was reported by Saudi cities, and the author divided them into five major regions (Central, East, South, West, and North).

### Primary end points

The study outcome was whether a patient died, and the date of the reported death. The overall mortality was defined as the percentage of patients who had died during the 45 days following the MERS-CoV diagnosis. The main outcome measures used were 3- and 30-day survival rates from the initial reported time of the MERS-CoV diagnosis to the reported death. However, the deaths were announced on the SMOH website without reporting the causes. Between December 2, 2014 and November 12, 2016, a total of 660 confirmed cases were reported by the Saudi Ministry of Health and have been included in this study.

## Statistical analysis

**Fig. 1:**
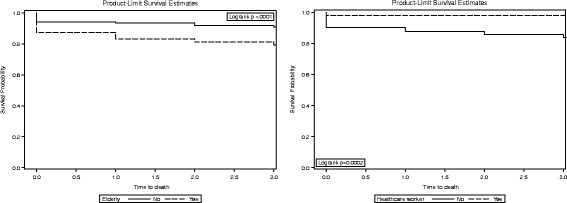
3-day survival by elderly and healthcare workers in MERS-CoV patients

**Fig. 2:**
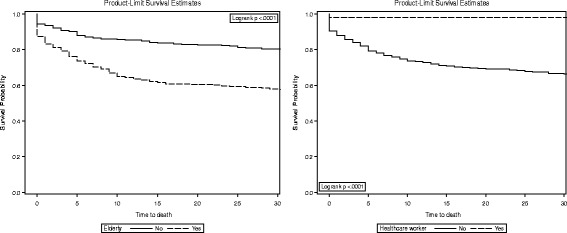
30-day survival by elderly and healthcare workers in MERS-CoV patients

**Fig. 3:**
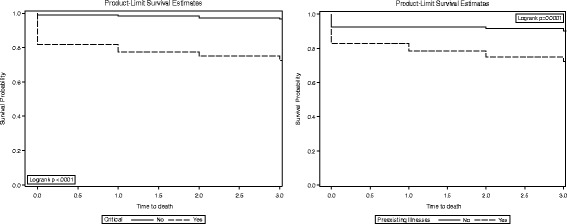
3-day survival by severity of illness and pre-existing illness in MERS-CoV patients

**Fig. 4:**
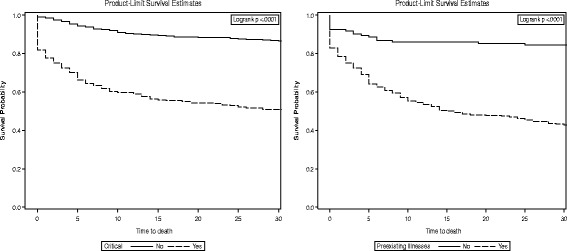
30-day survival by severity of illness and pre-existing illness in MERS-CoV patients

SAS version 9.4 (SAS Institute Inc*.,* Cary*,* NC*,* USA) was used to conduct the analyses. A summary of sample characteristics was reported in Table 1. Bivariate analyses: Chi-Square/Fisher’s exact tests were used to compare risks of overall mortality in MERS-CoV patients across the sample characteristics (Table 2). Cox-proportional hazard (CPH) models were applied to estimate the unadjusted hazard ratios for the predictors of 3- and 30-day mortality (Table 3). Multivariate analyses: Cox-proportional hazard (CPH) models were applied to estimate the adjusted hazard ratios for the predictors of 3- and 30-day mortality (Table 2). Included in the multivariate analyses were the following variables: age, gender, nationality, whether healthcare worker, pre-existing illness, severity of illness, and MERS-CoV source of infection. Furthermore, time to death was analyzed in terms of 3 days and 30 days by the Kaplan-Meier method (Figs. 1, 2, 3 and 4). The hypothesis test was two-tailed, with a *p* ≤ 0.05 indicative of statistical significance.

**Table 1:** MERS-CoV patients’ characteristics, December 2, 2014 - November 12, 2016

Characteristics	Number	Percent
Elderly	259	39.2
Male gender	452	68.6
Saudi nationality	466	70.6
Healthcare worker	105	15.9
Pre-existing illness	264	68.6
Symptomatic	621	94.4
Severity illness	276	41.9
Source of infections		
Camels	274	41.6
Household contact	44	6.7
Hospital-acquired infections	84	12.8
Unknown	256	38.9
Region		
Central	425	64.4
East	101	15.3
South	48	7.3
West/North	86	13
Outcome measures		
Overall mortality	197	29.8
3-day mortality	91	13.8
30-day mortality	187	28.3

**Table 2:** Overall mortality and its relation to patients’ characteristics, December 2, 2014 and November 12, 2016

		No		Yes		
Characteristics	Levels	463(70.2%)		197(29.8%)		*P*
Elderly	Yes	142	54.8	117	45.2	0.001*
	No	321	80.0	80	20.0	
Male Gender	Yes	311	68.8	141	31.2	0.281
	No	151	72.9	56	27.1	
Saudi Nationality	Yes	299	64.2	167	35.8	0.001*
	No	164	84.5	30	15.5	
Healthcare worker	Yes	103	98.1	2	1.9	0.001*
	No	360	64.9	195	35.1	
Pre-existing illness	Yes	104	39.4	160	60.6	0.001*
	No	102	84.3	19	15.7	
Symptomatic	Yes	427	68.8	194	31.2	0.001*
	No	36	97.3	1	2.7	
Severity of illness	Yes	138	50.0	138	50.0	0.001*
	No	325	85.1	57	14.9	
Source of infections	Camels	205	74.8	69	25.2	0.001*
	House hold contact	42	95.5	2	4.5	
	Hospital-acquired infections	61	72.6	23	27.4	
	Unknown	155	60.5	101	39.5	
Region	Central	293	68.9	132	31.1	0.401
	East	69	68.3	32	31.7	
	South	34	70.8	14	29.2	
	West/North	67	77.9	19	22.1	

*Significant at α = 0.05

**Table 3:** Estimated Hazard Risks of 3- and 30-day mortality for MERS-CoV patients

	3-day mortality 91(13.8%)								30-day mortality 187(28.3%)							
	*P*	HR	95% CI		*P*	aHR	95% CI		*P*	HR	95% CI		*P*	aHR	95% CI	
Elderly	0.001*	2.4	1.55	3.57	0.041*	1.7	1.02	2.71	0.001*	2.5	1.86	3.33	0.003*	1.7	1.18	2.31
Male gender	0.530	1.2	0.73	1.82	0.802	1.1	0.65	1.74	0.166	1.3	0.91	1.73	0.639	1.1	0.77	1.53
Saudi nationality	0.112	1.5	0.91	2.46	0.068	0.6	0.29	1.05	0.001*	2.5	1.66	3.66	0.168	0.7	0.44	1.15
Symptomatic	0.093	5.4	0.76	38.92	0.861	1.2	0.13	11.08	0.011*	12.6	1.77	90.14	0.281	3.1	0.40	24.07
Non-healthcare worker	0.002*	8.8	2.17	35.71	0.009*	8.8	1.73	44.62	0.001*	20.7	5.15	83.44	0.001*	19.2	4.37	84.68
Pre-existing illness	0.001*	3.0	1.61	5.44	0.256	1.5	0.76	2.77	0.001*	4.7	2.89	7.52	0.005*	2.1	1.25	3.39
Severity of illness	0.001*	8.9	4.94	16.04	0.001*	6.5	3.46	12.28	0.001*	4.7	3.43	6.55	0.001*	3.7	2.60	5.21
Source of infections																
Camels	0.023*	0.6	0.35	0.93	0.534	0.9	0.52	1.41	0.006*	0.6	0.47	0.88	0.725	0.9	0.67	1.32
Hospital-acquired infections	0.364	1.3	0.74	2.24	0.001*	2.8	1.49	5.22	0.255	0.8	0.49	1.21	0.001*	2.9	1.74	4.88
Household contact	0.042*	0.1	0.02	0.93	0.393	0.4	0.05	3.17	0.002*	0.1	0.03	0.43	0.096	0.3	0.07	1.24
Regions of Saudi Arabia																
Central	0.577	1.2	0.62	2.36	0.647	0.8	0.36	1.88	0.157	1.4	0.87	2.34	0.906	1.0	0.51	1.83
East	0.688	1.2	0.53	2.66	0.456	0.7	0.26	1.85	0.206	1.5	0.81	2.62	0.963	1.0	0.47	2.06
South	0.624	1.3	0.49	3.35	0.696	1.2	0.43	3.59	0.279	1.5	0.73	2.96	0.226	1.7	0.73	3.76

*Significant at α = 0.05. *HR* unadjusted estimates of hazard ratios, *aHR* adjusted estimates of hazard ratios

## Results

A total of 660 MERS-CoV infections in confirmed cases was reported by the Saudi Ministry of Health between December 2, 2014 and November 12, 2016. Of these, 39.2% were elderly (aged ≥60 years). The sample was relatively old, with a mean age of 53.9 (±SD 17.9) and with ages ranging between 2 and 109 years. More than two-thirds (68.6%) of the sample were males, 15.9% were healthcare workers, 68.6% had pre-existing illnesses, and 41.9% had severe illness conditions. Camel exposure was found in 41.6% of the cases. The majority (64.4%) of the cases were reported in the Central region of Saudi Arabia. Refer to Table 1 for the sample characteristics.

The subgroup analyses are shown in Table 2. Of the cases studied, 197 died with an overall mortality rate of 29.8% and a 95% confidence interval between 21.77%–28.53%. No significant difference in the overall mortality rate was noted between males and females (31.2% vs. 27.1%; *p* = 0.281). However, there was a significantly higher mortality rate in elderly patients (≥ 60 years) compared with patients aged <60 years (45.2% vs. 20.0%; *p* = 0.001). A higher mortality rate was found in those of the Saudi nationality (35.8% vs. 15.5% non-Saudi nationality; *p* = 0.001), those with preexisting illness (60.6% vs. 15.7% non-preexisting illness; *p* = 0.001), and the severity of illness (50.0% vs. 14.9% stable condition; *p* = 0.001). It was found that healthcare workers had a significantly lower mortality rate (1% vs. 29.6% non-healthcare workers; *p* = 0.001). A high risk of mortality was noted in patients with unknown (39.5%) sources of infections as compared to 4.5% household contact, 25.2% camels, 27.4% healthcare acquired; *p* = 0.001. The West/North region reported the lowest mortality rate (22.1%) as compared to 29.2% South, 31.7% East, 31.1% Central; *p* = 0.026.

The multivariate risk factors are shown in Table 2. During the 3 days of follow-up, 91 (13.8%) MERS-CoV patients died. Cox proportional hazard multivariate analysis identified several independent predictors of 3-day mortality in MERS-CoV patients. Elderly, non-healthcare worker, severity of illness, and hospital-acquired infections (adjusted hazard ratio [aHR]: 1.7; 8.8; 6.5; and 2.8, respectively). During the 30 days of follow-up, 187 (28.3%) MERS-CoV patients died. The 30-day mortality rate was higher in the elderly, non-healthcare worker, severity of illness, pre-existing illness, and hospital-acquired infections (aHR: 1.7; 19.2; 2.1; 3.7, and 2.9, respectively). The Kaplan–Meier method (Figs. 1 and 2) shows the 3-day (79.15% vs. 90.77%) and 30-day (57.92% vs. 80.55%) survival rates in the elderly group as compared to those of the under age 60 group. The 3-day (83.96% vs. 98.10%) and 30-day (66.67% vs. 98.10%) survival rates were in the non-healthcare worker group as compared to the healthcare worker group (Figs. 1 and 2).

## Discussion

The study results are based on 660 confirmed MERS-CoV infection cases extracted from public situational reports available on the Saudi Ministry of Health website. The study included cases from Saudi Arabia, and as of 27 February 2017, there have been reports of sporadic cases by the Saudi Ministry of Health. This data was also publicly available through the WHO website along with other MERS-CoV new case situational reports from other countries. Although the overall mortality rate (29.8%) in this study slightly decreased from what had been reported in Saudi Arabia, 60% [4], 42% [5], 40% [6, 7], 38% [8], with approximately one death in three of those infected with the MERS-CoV virus, it is still troubling and remains high when compared to South Korea [11, 12]. The recent declining in MERS-CoV mortality in Saudi Arabia could be due to implementation of prevention and control policies and placing great attention on MERS-CoV management in order to improve patient clinical outcomes. It was noted that there was a decline in the incidence of MERS-CoV infection reported over the past 2 years. A prior study has indicated that cases occurring later in the MERS-CoV emergence were less of a serious threat [18].

Little is known on 3- and 30-day survival of MERS-CoV patients. The study investigated 3- and 30-day survival rates and their risk factors. The 3- and 30-day survival rates in MERS-CoV patients were found to be 86.2% and 71.7%, respectively.

Mortality occurs more often in elderly patients (45.2%) than in those under age 60 (20.0%). Being elderly was associated with a 1.7 times higher risk of 3- and 30-day mortality as compared with those under age 60. The hazard of death in the elderly with MERS-CoV infections was 70% higher than with those under age 60. This evidence is consistent with most of previous studies, supporting the fact that older age is associated with increased risk of mortality in MERS-CoV patients [13–19]. Since it is not clear whether MERS-CoV related-death can be prevented among elderly patients, medical investigation and screening are warranted in elderly MERS-CoV patients to identify the reasons behind the high risk of death. The study findings suggest that there was no statistically significant difference in the 3- and 30-day survival between males and females, unlike other studies that suggest males have a higher risk of death [19, 20], while another study contradicts these two studies’ findings and suggest that males have a low risk of death [21]. Gender influence on 3- and 30-day survival is warranted, especially in the older age group.

A pre-existing illness was found to have an important effect on the risks of 30-day mortality in MERS-CoV patients (Figs. 3). The hazard of 30-day mortality in MERS-CoV patients with pre-existing illnesses is two times higher than in MERS-CoV patients without pre-existing illnesses. The 30-day survival rates were found to be 42.91% in MERS-CoV patients with pre-existing illness compared to 82.76% in MERS-CoV patients without pre-existing illness. Several studies confirmed, in agreement with the current study findings, an increased risk of death in patients with underlying comorbidity [14, 18, 19]. When necessary, a routine check-up and treatment of MERS-CoV patients with underlying comorbidity may be effective for MERS-CoV infection recovery and prevention of 30-day mortality.

MERS-CoV patients with severe illness conditions had a higher risk of 3- and 30-day mortality compared to patients without severe illness conditions (Fig. 3 and 4), which is also reflected in lower survival probability (72.46% vs. 96.60%) and (51.45% vs. 86.65%), respectively. When compared to the healthcare worker group, it was found that the non-healthcare worker group was associated with a 8.8- to 19.2-fold increase in the hazard of 3- and 30-day mortality. This is consistent with several previous studies [13, 18, 20, 21]. The low risk of death among the healthcare worker group could be due to timely and early diagnosis of MERS-CoV infection and the level of control and practices [22]. However, a large epidemiological study is needed to evaluate the high risk of death among the non-healthcare worker group. In such a study, modifiable factors could be assessed. For example, those can be addressed by the health system, such as access to healthcare, or by educational programs such as raising the level of awareness on proper management of infection and prevention practices.

The author noted several limitations. The study data was based on Saudi Ministry of Health situational reports which are publicly available sources and not directly aggregated from patients’ medical records or follow-ups. The date from symptoms onset was not reported publicly by the Saudi Ministry of Health, thus the time of the MERS-CoV diagnosis to outcome was used, rather than the date of symptom onset. Not all patients experience MERS symptoms, while other patients may experience mild or severe respiratory illness, thus the survival rate may differ if the date of symptom onset was used. A delay in the MERS diagnosis could worsen patient outcome and increase the risk of death. The patient outcomes (died/alive) and new cases are updated daily by the Saudi Ministry of Health. Thus, the patient outcomes may not represent the final outcomes. The new cases, time of diagnosis, death status, and date of death were recorded at the time of reporting. However, the current analyses were based on the report as of November 12, 2016, where the data has been accessed by the study author. As this data is gathered from public sources, there is no information on cause of death or details on underlying conditions. Not having the specific underlying conditions at hand, the current study findings suggest that this type of association might become a prerequisite for interventional studies. There is no information regarding patients’ occupations, however it may be that there are possible links between occupation and MERS-CoV infection. Nonetheless, the current study evaluated MERS-CoV outcomes, such as 3- and 30-day mortality and their risk factors. A great advantage of the conducted study is that the author extracted large MERS data and took several prognostic factors into account to predict 3- and 30-day mortality in MERS. This study could be of value to policymakers and healthcare systems worldwide in identifying patients with high risk of death and shorter survival. The study revealed several prognostic factors that might explain the high rates of early mortality. It looks promising (in both bivariate and multivariate analyses) that having such information was associated with 3- and 30-day mortality. The results of this study may be useful to healthcare professionals who are providing medical care for MERS patients by appropriately identifying and managing potential patients at high risk of death.

## Conclusion

Several factors were identified that could influence mortality outcome at 3 days and 30 days, including being elderly, being a non-healthcare worker, along with illness severity and hospital-acquired infections. The findings can serve as a guide for healthcare practitioners when monitoring and managing the virus, especially at the early stages. Implementing outcome measures and strategies to improve patients’ survival in MERS-CoV patients should be an ultimate goal for public health policymakers.

## Abbreviations

aHR: Adjusted hazard ratio
CCC: Command and control center
CPH: Cox-proportional hazard
MERS-CoV: Middle east respiratory syndrome coronavirus
SMOH: Saudi Ministry of Health
WHO: World Health Organization
